# Interrelation between weight and weight stigma in youth: is there evidence for an obesogenic vicious cycle?

**DOI:** 10.1007/s00787-021-01922-3

**Published:** 2021-12-01

**Authors:** Michaela Silvia Gmeiner, Petra Warschburger

**Affiliations:** Department of Psychology, University of Potsdam, Karl-Liebknechtstr. 24-25, 14476 Potsdam, Germany

**Keywords:** Weight, Stigma, Children, Adolescents, Structural equation modelling

## Abstract

Many children and adolescents are confronted with weight stigma, which can cause psychological and physical burden. While theoretical frameworks postulate a vicious cycle linking stigma and weight status, there is a lack of empirical evidence. The aim was to analyze the longitudinal bidirectional relationship between body weight and weight stigma among children and adolescents. The sample consisted of 1381 children and adolescents, aged 9–19 years at baseline (49.2% female; 78% normal weight), from a prospective study encompassing three measurement points over 6 years. Participants provided self-reported data on experienced weight-related teasing and weight/height (as indicators for weight status). Latent structural equation modelling was used to examine the relationship between weight-related teasing experiences and weight. Additionally, gender-related differences were analyzed. Between the first two waves, there was evidence for a bidirectional relationship between weight and weight-related teasing. Between the last two waves, teasing predicted weight, but there was no reverse association. No gender-related differences were found. The data indicate a reciprocal association between weight stigma and body weight across weight groups and independent of gender. To prevent vicious cycles, approaches that simultaneously promote healthy weight and reduce weight stigma are required.

## Introduction

Being overweight is often associated in society with various negative attributes, such as being undisciplined, unmotivated, lazy, incompetent, ugly or unattractive. Prevailing stereotypes and prejudices are common and also lead to weight discrimination: those categorized as too heavy are likely to face devaluation and unfair treatment [1, 2]. In youth, weight stigma often manifests as weight-related bullying and teasing. Even though this stigma is more prevalent among individuals with overweight, it is a widespread phenomenon that affects children and adolescents across all weight categories [1–3]; up to 50% of youth have reported weight-related bullying [3]. Negative health consequences of weight stigma are well-documented and include psychosocial problems (e.g., depressive or anxious symptoms, decreased self-esteem and body image, maladaptive eating behaviors, reduced physical activity, substance abuse), as well as physical impairments (e.g., increased risk for cardiovascular disease) [1–3].

Given the fact that cross-sectional studies show that weight stigma is associated with a higher risk for overweight or obesity among youth [4], theoretical frameworks postulate a vicious cycle between weight stigma and weight [5, 6]. Indeed, there is a rationale for the assumption that stigma reinforces weight gain, as well as the reverse [4]. Frequent exposure to weight stigma might increase weight or maintain obesity as a result of various emotional, cognitive, behavioral or physical mechanisms (for details, see [1, 6–9]). Similarly, higher weight status might result in increased or persisting weight stigma, because, for example, the stigmatized feature becomes more apparent [10] or stigmatization is seen as a legitimate motivator for weight loss [7]. A recent meta-analysis concluded that initial prospective studies (*n* = 5) provide evidence for both pathways among children and adolescents [4]. So far, only one study [11] simultaneously considered the bidirectional relationship between weight stigma and body mass index (BMI), but only among boys. The majority of the studies examined monodirectional effects only. This might result in spurious effects and therefore an overestimation of the relationship between weight stigma and weight [12]. There is a lack of evidence for the proposed reciprocal reinforcement between weight and weight stigma in the literature. Therefore, the major aim of our study was to analyze the prospective bidirectional relationship between weight and weight stigma. We hypothesized that weight-related stigma would predict future weight and vice versa.

In the context of weight stigma, potential gender differences are discussed. Whereas some studies observed higher perceived weight-related stigma among female children compared to male ones or vice versa, others found no differences [3, 13]. A recent meta-analysis reported similar cross-sectional correlations between BMI and weight stigma across genders [4]. With regard to prospective studies, three studies did not observe gender differences with regard to the effect of weight-related stigma on weight [11, 14] or vice versa [15]. Contrary, other studies reported that previous experience of weight stigma came along with a higher risk for obesity in girls, but not in boys [16], and that the predictive effect of weight stigma for BMI was higher among girls compared to boys [17]. Overall, prospective studies concerning gender differences are still sparse and do not allow meta-analytic pooling [4]. At this point, there is still no study that directly compares longitudinal reciprocal effects between weight and weight-related stigma across genders. Therefore, the second aim of this study was to compare the prospective bidirectional relationship between weight and weight stigma across genders in an exploratory manner.

## Methods

### Procedure

Data were derived from the PIER study, a large population-based prospective study on intrapersonal developmental risk factors in childhood and adolescence. Participants were recruited from various areas in the Federal State of Brandenburg, Germany, and written and informed consent and assent were obtained. Data were assessed at three points in time. The interval between the first (T1, 2011–2012) and second (T2, 2013–2014) assessments was approximately 20 months (*M* = 19.97, *SD* = 3.99); the third assessment (T3, 2016) followed an additional 30 months later (*M* = 29.97, *SD* = 3.25).

T1 and T2 were carried out by trained research assistants. The individual sessions included tests and self-report questionnaires (completed on a computer screen or as a paper–pencil version), took around 90–120 min each and were conducted at school, the family’s home or in the university's facilities. For logistical reasons, at T3 questionnaires were completed at home (online or paper–pencil version; approximate duration: 60 min); participation in the additional face-to-face tests was optional. For each wave, vouchers for cinemas or online shops (10€) were provided to reward participation. The study was approved by the local ethics committee.

### Sample characteristics

The final sample consisted of 1,381 children and adolescents between 9 and 19 years of age (*M* = 13.35, *SD* = 2). Participants who failed to provide self-reported anthropometric data (*n* = 106) or self-reported weight teasing (*n* = 2) at least once during measurement procedures were excluded from analyses. The participation rate across genders was balanced (49.2/50.4/55.3% females at T1/T2/T3). Educational background was inquired via parental education level: 46.7% stated a higher education (e.g., BA, MA, Diploma, PhD etc.); 11% reached higher education entrance qualifications (‘Abitur,’ equivalent of a high school degree/A-level) and 24.5% reported secondary school graduation or below; 17.8% did not provide information about their education level. Further descriptive data can be found in Table 1.

**Table 1:** Descriptive statistics at times 1 (T1), 2 (T2) and 3 (T3)

		T1 (*n* = 1381)			T2 (*n* = 1078)			T3 (*n* = 624)		
		Overall	Female	Male	Overall	Female	Male	Overall	Female	Male
Age	*M* (*SD*)	13.35 (2)	13.41 (1.99)	13.29 (2.01)	14.78 (1.92)	14.92 (1.96)	14.64 (1.87)	17.02 (1.92)	17.15 (1.95)	16.84 (1.89)
	Range	9.93–19.45	9.96–18.51	9.93–19.45	11.29–20.53	11.38–20.37	11.29–20.53	14.08–22.75	14.08–22.42	14.08–22.75
Weight (status)^1^	BMI-SDS *M* (*SD*)	− 0.13 (1.09)	− 0.17 (1.05)	− 0.09 (1.13)	− 0.03 (1.08)	− 0.15^a^ (1.06)	0.08^a^ (1.1)	− 0.16 (0.99)	− 0.22 (0.99)	− 0.09 (0.98)
	% (n) underweight	13.9 (187)	14 (92)	13.8 (95)	11.3 (119)	12.2 (65)	10.4 (54)	13.5 (83)	14.6 (50)	12.2 (33)
	% (n) normal weight	77.9 (1049)	78.6 (518)	77.2 (531)	77.6 (817)	78.7 (420)	76.5 (397)	80.1 (491)	80.1 (274)	80.1 (217)
	% (n) overweight	8.2 (111)	7.4 (49)	9 (62)	11.1 (117)	9.2 (49)	13.1 (68)	6.4 (39)	5.2 (18)	7.7 (21)
Weight-related teasing^2^	*M* (*SD*)	1.13 (0.36)	1.13 (0.36)	1.13 (0.36)	1.11 (0.3)	1.12 (0.32)	1.09 (0.29)	1.07 (0.24)	1.09^b^ (0.29)	1.03^b^ (0.14)

*BMI-SDS* body mass index standard deviation score

Descriptive values are based on raw data (not imputed)

^1^Subjective weight data were available for 97.5% (*n* = 1347) of cases at T1/76.2% (*n* = 1053) at T2/44.4% (*n* = 613) at T3. Based on national reference data, underweight was defined as BMI < 10th percentile, overweight as BMI > 90th percentile

^2^Data about weight-related teasing were available for 99.9% (*n* = 1379) of cases at T1/78.1% (*n* = 1078) at T2/45% (*n* = 621) at T3)

Significant gender differences are indicated by superscripted letters:

^a^(*t*(1051) = 3.41, *p* = 0.001, *d* = 0.21)

^b^(*t*(497) = 3.18, *p* = 0.002, *d* = 0.24)

Data across all points of measurement were available for 45% (*n* = 624); 33% (*n* = 454) of participants took part in two of the three assessments, 22% (*n* = 303) in one of the three. Attrition rate between T1 and T2 was 21.9% and 42.1% from T2 to T3. Changes in procedure and a lower availability of older participants (e.g., due to changes of school or leaving the parental home) might have led to the relatively high attrition rate in the last wave. Systematic comparisons revealed that non-participants at T2 and T3 were older (T2: *t*(1379) = 8.35, *p* < 0.001, *d* = 0.54; T3: *t*(1379) = 8.03, *p* < 0.001, *d* = 0.43). Moreover, non-participants at T3 were heavier (*t*(1345) = 3.97, *p* < 0.001, *d* = 0.22), were more often males (χ^2^ = 17.07, *p* < 0.001, $\phi$ = 0.11) and reported more frequent weight-related teasing (*t*(1328) = 3.32, *p* = 0.001, *d* = 0.17) at baseline.

## Materials and measures

### Body weight

Based on national reference data, we calculated body mass index standard deviation scores (BMI-SDS) from self-reported weight and height [18, 19]. Additionally to self-reports, weight and height were also measured objectively by research staff. However, at T3 objective weight measurements could only be collected for a few cases (29.7%) due to the voluntary nature of the additional on-site measurements. Accordingly, our analyses refer to subjective data to keep the method consistent across the measurement points. Correlation with objective BMI-SDS was high across all measurement points (*r*_T1_ = 0.82 [*n* = 1267]; *r*_T2_ = 0.84 [*n* = 983]; *r*_T3_ = 0.93 [*n* = 404]).

### Weight-related teasing

Weight-related teasing was assessed with five items from the perception of teasing scale (POTS [20]), which was adapted for children. Children and adolescents rated its frequency (e.g., “How often do people laugh at you because you are heavy?”) on a 5-point scale (1 = *never* to 5 = *very often*). In the current sample, internal consistencies were high (α_T1_ = 0.86, α_T2_ = 0.83, α_T3_ = 0.83).

### Data analyses

**Fig. 1:**
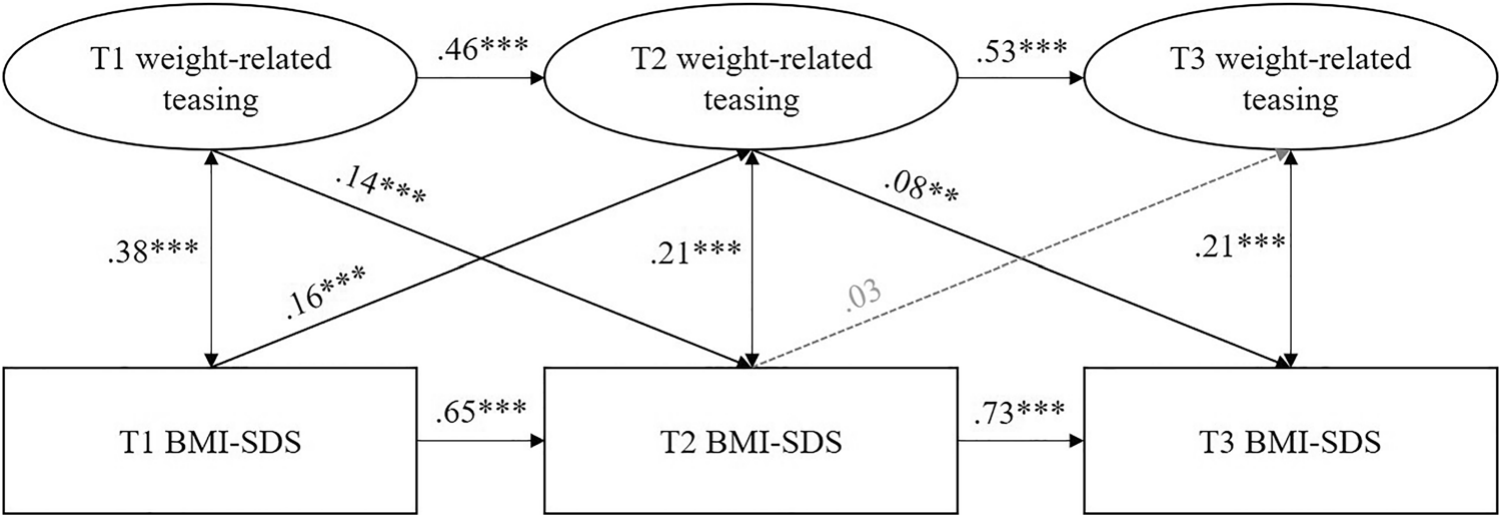
Prospective relationships between weight (BMI-SDS) and weight-related teasing for children and adolescents. Standardized results are reported. Variables are controlled for age and gender at the respective point of measurement.* T1/T2/T3* point of measurement 1/2/3,* BMI-SDS* body mass index standard deviation score. Grey dotted lines indicate non-significant results (*p* > 0.05). ***p < 0.001, **p < 0.01

Statistical analyses were performed with IBM SPSS Statistics 27 (descriptive data, drop-out-analyses) and MPLUS 7.0 (latent path modelling). We used structural equation modelling to test the theoretical model (displayed in Fig. 1). Our preceding analyses ensured that the POTS showed adequate measurement invariance across time and genders. Therefore, teasing was considered as a latent variable. BMI-SDS was included as a manifest variable. We included autoregressive paths to control for stability and allowed cross-sectional correlations between the variables. All variables were controlled for age and gender. To test for differences across gender, we conducted multigroup comparisons. Wald tests were applied to compare path coefficients. We evaluated the model data fit as follows: a good fit was indicated by a non-significant χ^2^, χ^2^/df ratio ≤ 3, root mean square error of approximation (RMSEA) ≤ 0.05, comparative fit index (CFI) ≥ 0.95 and root mean square residual (SRMR) ≤ 0.1 [21]. For all analyses, the alpha level was determined at *p* < 0.05. We employed full information maximum likelihood (FIML) estimation to cope with missing data on single variables and drop-outs. This model-based procedure is recommended, as it is superior to other techniques [22] and reduces the risk of biased results caused by the list-wise depletion of drop-outs. Due to non-normality of the POTS items, we applied robust maximum likelihood estimators (MLR) [23].

## Results

Descriptive data on weight-related teasing and BMI-SDS are displayed in Table 1.

### Prospective relationships between BMI-SDS and weight-related teasing

The proposed model showed a good fit to the data (χ^2^(191) = 300.98, *p* < 0.001; χ^2^/df = 1.58; RMSEA = 0.02; CFI = 0.98; SRMR = 0.06). Results are displayed in Fig. 1. As expected, significant cross-sectional associations were observed between weight-related teasing and BMI-SDS at each measurement point, with the highest correlation at T1 (*β* = 0.28, *p* < 0.001) and slightly lower correlations at T2 and T3 (*β* = 0.21, *p* < 0.001). With regard to the autoregressive paths, BMI-SDS (*β*_T1–T2_ = 0.65, *β*_T2–T3_ = 0.73; *p* < 0.001) and weight-related teasing (*β*_T1–T2_ = 0.46, *β*_T2–T3_ = 0.53; *p* < 0.001) showed a high stability. As expected, weight-related teasing and BMI-SDS at T1 predicted each other at T2. Weight-related teasing at T2 in turn predicted BMI-SDS at T3, whereas the reciprocal association was not observed. Variance in all endogenous variables was explained significantly (perception of weight-related teasing: 29.1% at T2, 28.6% at T3; BMI-SDS: 50.9% at T2, 58.2% at T3; *p* < 0.05).

Additionally, analyses were rerun with objectively measured weight data. Due to the low rate of objective measurements at T3 (< 30%), we refrained from imputing these data. These exploratory analyses delivered quite comparable results, although cross-lagged paths between T2 and T3 were only marginally significant.

### Multigroup comparison across genders

**Fig. 2:**
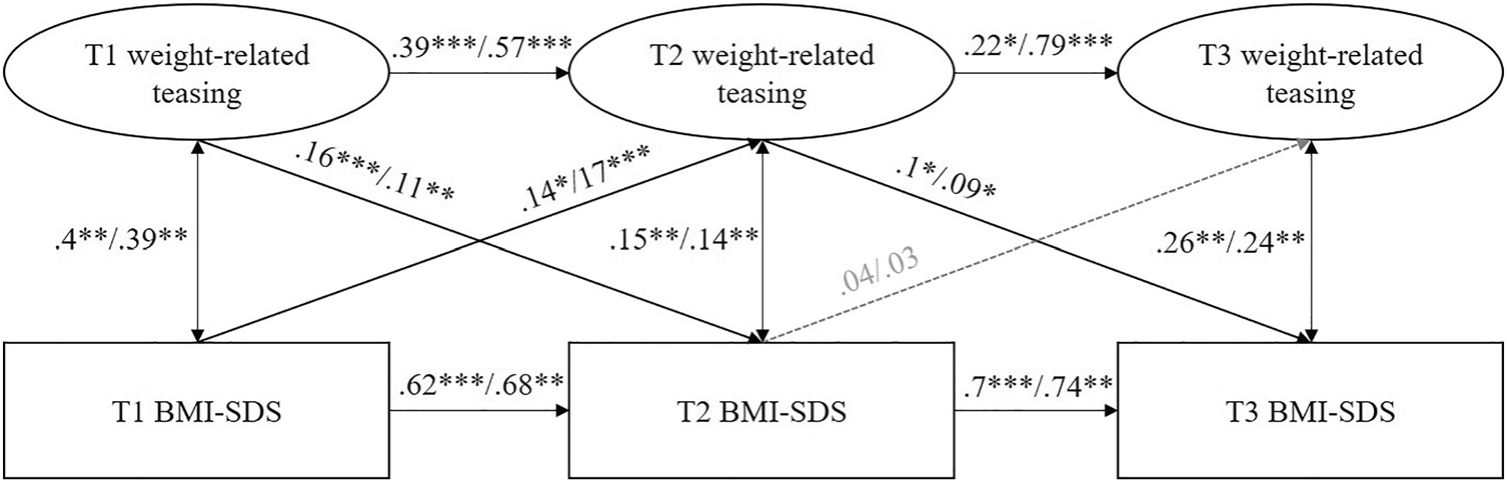
Prospective relationships between weight (BMI-SDS) and weight-related teasing for males and females. Standardized path coefficients for male/female children and adolescents. Variables are controlled for age at the respective point of measurement.* T1/T2/T3* point of measurement 1/2/3,* BMI-SDS* body mass index standard deviation score. Grey dotted lines indicate non-significant results (*p* > 0.05). ***p < 0.001, **p < .01, *p < 0.05

The model of the multigroup comparison showed an acceptable fit to the data (χ^2^(191) = 609.22, *p* < 0.001; χ^2^/df = 1.58; RMSEA = 0.03; CFI = 0.96; SRMR = 0.14). On the whole, results were comparable to those of the total sample (see Fig. 2). The Wald test revealed no significant differences between male and female children and adolescents, except that females reported a higher stability of weight-related teasing (*W*_T1–T2_ = 3.51, *p* = 0.061, *W*_T2–T3_ = 5.58, *p* = 0.018).

## Discussion

To sum up, the cross-lagged panel analyses revealed a reciprocal relationship between weight-related teasing and weight in children and adolescents across all weight groups. More specifically, higher weight-related teasing was consistently associated with higher future BMI-SDS, and BMI-SDS also predicted weight-teasing; however, this was only observed between the first two measurements. We did not find evidence for differences across genders.

Corresponding with previous findings [4], weight-related teasing and weight were not only correlated cross-sectionally but also predicted each other longitudinally. Accordingly, the results indicate a cyclical relationship between weight stigma and weight, although this was not consistent across all points of measurement. Two previous studies reported a longitudinal influence of weight on weight stigma in children and adolescents [11, 15]. We can only speculate as to why BMI-SDS at T2 did not predict weight-related teasing at T3 in the current sample. For one thing, the temporal distance between T1 and T2 (approximately 20 months) was shorter than between T2 and T3 (approximately 30 months). This complicates the clear interpretation of bidirectional effects but might suggest that weight could be predictive of weight stigma only over relatively shorter time periods. However, the design allows no conclusion about underlying mechanisms or individual weight fluctuations between the measurements. These should be examined more in-depth by future studies (e.g., on the basis of shorter measurement intervals).

Besides this, participants were getting older during the course of the survey. Increasing age is associated with a higher acceptance of various body sizes deviating from the thin ideal [24, 25]. This could mean that weight is no longer predictive for weight stigma among older participants. Further, the missing link may result from our operationalization focusing on weight-related teasing defined as laughing at individuals or joking about heavy weight. Even so, the nature of weight stigma might differ by age so that, for older individuals at the last point of measurement, other forms of weight stigma (e.g., social exclusion) might have been more relevant [26]. Ultimately, however, due to overlapping age spans across waves, no conclusion as to the age effect was possible in our study. Accordingly, a comparison of age groups could be of further interest [27].

With respect to gender differences, we observed a lower stability of teasing in male (compared to female) children and adolescents between the last two measurements. This is consistent with a study showing that weight teasing overall might be relatively stable and track into young adulthood, but decreases in males (but not females) from early adolescence to young adulthood [28]. Beyond that, it should be mentioned that we did not observe gender-related differences with respect to the relationship between weight-related stigma and BMI-SDS—neither cross-sectional nor longitudinal. This is in line with previous studies that also reported no gender differences [14, 15], but runs contrary to others indicating that there might be gender differences [16, 17]. A closer examination reveals that moderators might explain inconsistent results regarding gender differences. For one thing, consideration of the perpetrators of teasing might be an essential variable: among females, weight increased when being teased by either families or peers, whereas among males only teasing by peers had a significant contribution to the further weight course [17]. Additionally, one study reported that weight-related teasing predicted future overweight only for girls, but an increase in teasing over time was predictive of future overweight among both genders [16]. Future studies examining and comparing the bidirectional associations among males and females are warranted. This would help explain the complex association between body weight and weight stigma in more detail.

Taken together, our study underscores that weight and weight-related teasing reinforce each other over the long term. As a consequence, experiencing weight stigma or being heavier might lead to a vicious cycle—with the result that both will worsen. Weight-related stigma as well as being overweight are associated with various detrimental negative health outcomes [1–3, 29, 30]. These in turn might also be intensified, and negative consequences could persist into adulthood [31]. This highlights the importance of interrupting such vicious cycles to prevent adverse trajectories. Preventive and interventional strategies might be especially effective if they simultaneously address both levels: reduction of weight stigma as well as promoting healthy weight development to prevent future weight gain. Therefore, preventive public health approaches (e.g., health campaigns at schools), as well as health-promoting interventions, should focus on non-stigmatizing content and the promotion of weight-neutral healthy lifestyles instead of focusing on weight per se [7].

Presently, there are several assumptions about the underlying mechanisms that might explain the bidirectional relationship between weight and weight-related teasing [5–7], such as weight bias internalization, eating behavior, body image and coping. Knowledge derived from these studies might improve preventive approaches and interventions to stop the vicious cycle between weight and weight stigma.

### Strengths, limitations and future implications

This is the first study to analyze the cross-lagged longitudinal relationship between weight and weight-related stigma among youth and compare bidirectional relationships across gender. Nevertheless, there are some limitations that should be mentioned. First, there was a high and also systematic drop-out over time. We applied the FIML approach to take drop-outs into account, but that does not completely eliminate the risk of bias. As those who dropped out showed a higher BMI-SDS and experienced more frequent weight teasing at baseline, the role of weight teasing might be underestimated. Second, current weight-related teasing was assessed by the POTS as laughing or joking about weight, but other types of stigma which might vary across age spans [26], were disregarded. This allowed to keep the method consistent across the measurement points but might lead to an underestimation of other types of weight stigma that might become more relevant in older participants. Furthermore, the study design depicts the current individual evaluation of weight teasing but cannot provide information about fluctuations or underlying mechanisms within the interval between the measurement points. In addition, the widespread of ages at baseline impedes conclusions about the role of developmental stages in the relationship between weight and weight stigma and the timing of interventions to interrupt the cycle. As the current data were not suited to analyze appropriate age bands, future research could take care of this problem, e.g., by means of multi-cohort-sequence designs. Fourth, analyses were based on self-reported weight data. We exploratively reran the analyses with objective weight data and yielded similar results, but these were limited by a very small data base at T3. Although self-reported and objective anthropometric data were highly correlated [32], in general self-reported data underestimated the objective weight status (data not shown). This was acceptable, as we focused on the presence of relationships over time instead of interpreting absolute mean scores. As differences between self-reported and objective data were not associated with weight teasing (data not shown), we presume that a potential underestimation of effects is constant across time points and allows us to draw sound conclusions about the pattern of temporal relationships—even though the magnitude of these associations might be underestimated. Nevertheless, future studies on the basis of objective weight data are warranted. Last but not least, our sample size was not sufficient to compare the model between weight groups. Future studies should therefore examine whether the tested model differs across weight groups. We included individuals with normal and underweight, as there is evidence that weight stigma is prevalent across all weight groups [17]. Nonetheless, one might assume that the association between weight stigma and weight is especially relevant among those who are already overweight and should pay special attention to this group.

The present study also has several strengths. First, the prospective design with three points of measurement allowed an investigation of reciprocal effects over the course of six years. Second, the large sample with a balanced gender ratio allowed us to test complex models. Third, we implemented a latent modeling of weight-related teasing. Overall, this valuable methodological approach enabled us to control for random effects of measuring errors, to verify measurement invariance across time and genders, and to consider the stability of the included variables.

## Conclusions

In conclusion, weight and weight-related stigma were shown to be interrelated over the long term, leading to a vicious cycle for both females and males across weight groups. Effective approaches are needed to interrupt detrimental trajectories and comorbid negative health outcomes. Therefore, it might be promising to simultaneously address weight stigma reduction and healthy lifestyle interventions to either promote weight loss or hinder future weight gain. In addition, it might be crucial to identify factors that mediate or moderate the relationship between weight stigma and weight.

## Data Availability

The datasets generated and analyzed during the current study are not publicly available, as the participants were not asked to consent to publication within repositories, but are available from the corresponding author on reasonable request.

## References

[1] Pont SJ, Puhl RM, Cook SR (2017). Stigma experienced by children and adolescents with obesity. Pediatrics.

[2] Puhl RM, King KM (2013). Weight discrimination and bullying. Best Pract Res Clin Endocrinol Metab.

[3] Puhl RM, Lessard LM (2020). Weight stigma in youth: prevalence, consequences, and considerations for clinical practice. Curr Obes Rep.

[4] Ma L, Chu M, Li Y (2021). Bidirectional relationships between weight stigma and pediatric obesity: a systematic review and meta-analysis. Obes Rev.

[5] Tomiyama AJ (2014). Weight stigma is stressful. A review of evidence for the cyclic obesity/weight-based stigma model. Appetite.

[6] Brewis AA (2014). Stigma and the perpetuation of obesity. Soc Sci Med.

[7] Major B, Tomiyama AJ, Hunger JM, Major B, Dovidio JF, Link BG (2018). The negative and bi-directional effects of weight stigma on health. The Oxford handbook of stigma, discrimination, and health.

[8] Sikorski C, Luppa M, Luck T (2015). Weight stigma “gets under the skin”-Evidence for an adapted psychological mediation framework: a systematic review. Obesity.

[9] Puhl RM, Himmelstein MS, Pearl RL (2020). Weight stigma as a psychosocial contributor to obesity. Am Psychol.

[10] Griffiths LJ, Wolke D, Page AS (2006). Obesity and bullying: different effects for boys and girls. Arch Dis Child.

[11] Straatmann VS, Almquist YB, Oliveira AJ (2018). Cross-lagged structural equation models for the relationship between health-related state and behaviours and body bullying in adolescence: findings from longitudinal study ELANA. PLoS ONE.

[12] Kenny DA (1975). Cross-lagged panel correlation: a test for spuriousness. Psychol Bull.

[13] Puhl RM, Latner JD (2007). Stigma, obesity, and the health of the nation’s children. Psychol Bull.

[14] Schvey NA, Marwitz SE, Mi SJ (2019). Weight-based teasing is associated with gain in BMI and fat mass among children and adolescents at-risk for obesity: a longitudinal study. Pediatr Obes.

[15] Zuba A, Warschburger P (2017). The role of weight teasing and weight bias internalization in psychological functioning. A prospective study among school-aged children. Eur Child Adolesc Psychiatry.

[16] Quick V, Wall M, Larson N (2013). Personal, behavioral and socio-environmental predictors of overweight incidence in young adults: 10-yr longitudinal findings. Int J Behav Nutr Phys Act.

[17] Puhl RM, Wall MM, Chen C (2017). Experiences of weight teasing in adolescence and weight-related outcomes in adulthood. A 15-year longitudinal study. Prev Med.

[18] Kromeyer-Hauschild K, Moss A, Wabitsch M (2015). Referenzwerte für den Body-Mass- Index für Kinder, Jugendliche und Erwachsene in Deutschland. Anpassung der AGA-BMI-Referenz im Altersbereich von 15 bis 18 Jahren (Body mass index reference values for German children, adolescents and adults. Modification of the AGA BMI reference in the age range between 15 and 18 years). Adipositas.

[19] Kromeyer-Hauschild K, Wabitsch M, Kunze K (2001). Perzentile für den Body-Mass-Index für das Kindes- und Jugendalter unter Heranziehung verschiedener deutscher Stichproben (Percentiles of body mass index in children and adolescents evaluated from different regional German studies). Monatsschr Kinderheilkd.

[20] Thompson JK, Cattarin J, Fowler B (1995). The perception of teasing scale (POTS): a revision and extension of the Physical Appearance Related Teasing Scale (PARTS). J Pers Assess.

[21] Hu L, Bentler PM (1999). Cutoff criteria for fit indexes in covariance structure analysis: conventional criteria versus new alternatives. Struct Equ Modeling.

[22] Enders CK (2010). Applied missing data analysis.

[23] Kline RB (2016). Principles and practice of structural equation modeling.

[24] Rand CS, Wright BA (2000). Continuity and change in the evaluation of ideal and acceptable body sizes across a wide age span. Int J Eat Disord.

[25] Latner JD, Stunkard AJ, Wilson GT (2005). Stigmatized students: age, sex, and ethnicity effects in the stigmatization of obesity. Obes Res.

[26] Parker JG, Rubin KH, Erath SA, Cicchetti D, Cohen DJ (2006). Peer relationships, child development, and adjustment: a developmental psychopathology perspective. Developmental psychopathology. Volume one: Theory and method.

[27] Haines J, Neumark-Sztainer DR, Hannan PJ (2008). Longitudinal and secular trends in weight-related teasing during adolescence. Obesity.

[28] Haines J, Hannan PJ, van den Berg PA (2013). Weight-related teasing from adolescence to young adulthood: longitudinal and secular trends between 1999 and 2010. Obesity.

[29] Sharma V, Coleman S, Nixon J (2019). A systematic review and meta-analysis estimating the population prevalence of comorbidities in children and adolescents aged 5 to 18 years. Obes Rev.

[30] Rankin J, Matthews L, Cobley S (2016). Psychological consequences of childhood obesity: psychiatric comorbidity and prevention. Adolesc Health Med Ther.

[31] Simmonds M, Burch J, Llewellyn A (2015). The use of measures of obesity in childhood for predicting obesity and the development of obesity-related diseases in adulthood: a systematic review and meta-analysis. Health Technol Assess (Winchester, England).

[32] Brettschneider A-K, Rosario AS, Ellert U (2011). Validity and predictors of BMI derived from self-reported height and weight among 11- to 17-year-old German adolescents from the KiGGS study. BMC Res Notes.

